# Genetic delineation of local provenance defines seed collection zones along a climate gradient

**DOI:** 10.1093/aobpla/plv149

**Published:** 2016-01-11

**Authors:** Kristina M. Hufford, Erik J. Veneklaas, Hans Lambers, Siegfried L. Krauss

**Affiliations:** 1 School of Plant Biology, The University of Western Australia, Crawley, WA 6009, Australia; 2 Department of Ecosystem Science and Management, University of Wyoming, Laramie, WY 82071, USA; 3 Kings Park and Botanic Garden, Botanic Gardens and Parks Authority, West Perth, WA 6005, Australia

**Keywords:** AFLP, BayeScan, ecological restoration, southwestern Australia, spatial genetic structure, *Stylidium hispidum*

## Abstract

Efforts to re-establish native plant species should consider intraspecific variation if we are to restore genetic diversity and evolutionary potential. Data describing spatial genetic structure and the scale of adaptive differentiation are needed for restoration seed sourcing. Genetically defined provenance zones provide species-specific guidelines for the distance within which seed transfer likely maintains levels of genetic diversity and conserves locally adapted traits. While a growing number of studies incorporate genetic marker data in delineation of local provenance, they often fail to distinguish the impacts of neutral and non-neutral variation. We analysed population genetic structure for 134 amplified fragment length polymorphism (AFLP) markers in *Stylidium hispidum* (Stylidiaceae) along a north–south transect of the species' range with the goal to estimate the distance at which significant genetic differences occur among source and recipient populations in restoration. In addition, we tested AFLP markers for signatures of selection, and examined the relationship of neutral and putatively selected markers with climate variables. Estimates of population genetic structure revealed significant levels of differentiation (Φ_PT_ = 0.23) and suggested a global provenance distance of 45 km for pairwise comparisons of 16 populations. Of the 134 markers, 13 exhibited evidence of diversifying selection (Φ_PT_ = 0.52). Using data for precipitation and thermal gradients, we compared genetic, geographic and environmental distance for subsets of neutral and selected markers. Strong isolation by distance was detected in all cases, but positive correlations with climate variables were present only for markers with signatures of selection. We address findings in light of defining local provenance in ecological restoration.

## Abstract (precis)

Ecological restoration is often conducted with limited consideration of genetic diversity and the environmental factors that drive variation within species. We studied genetic diversity and environmental variation among 16 populations of *Stylidium hispidum*, an endemic southwestern Australian triggerplant. As a result we were able to estimate the seed transfer distance within which genetic divergence is low and population fitness less likely to be impacted by maladaptation, and identify environmental variables that may be relevant for future restoration of this species.

## Introduction

Ecological restoration is often conducted with limited consideration of genetic diversity or the environmental factors that influence intraspecific variation (Rice and Emery 2003; Bischoff *et al*. 2010; Byrne *et al*. 2011). Large-scale introductions of propagules can result in genetic bottlenecks if seeds are collected from a limited number of sources. Alternatively, wide mixing of provenances may result in negative consequences due to lower fitness of introduced plants, or outbreeding depression as a result of cross-pollination among differently adapted genotypes (Hufford and Mazer 2003). Efforts to re-establish native species should target evolutionary potential as well as ecological processes, and restoration programmes can benefit from knowledge of spatial genetic structure and the scale of adaptive differentiation in focal species (Broadhurst *et al*. 2008; Williams *et al*. 2014).

Data for intraspecific variation in adaptive traits are difficult to obtain for most native, non-commercial species, especially at the scale of large restoration programmes (Kawecki and Ebert 2004; Savolainen *et al*. 2013). As a result, seed sourcing guidelines for restoration are often limited to general ‘rules of thumb’ to conserve genetic diversity and match environmental conditions between donor and restoration sites (Knapp and Rice 1994; Lesica and Allendorf 1999; McKay *et al*. 2005). A growing number of studies aim to improve these guidelines through the use of genetic data to determine the scale of local adaptation and delineate species-specific seed provenance zones (e.g. Stingemore and Krauss 2013; Bower *et al*. 2014; Dillon *et al*. 2014). Marker-delineated provenance zones describe the radius within which seed transfer is predicted to limit the risk for population fitness by defining the distance beyond which significant genetic divergence among populations occurs (Krauss and Koch 2004).

While there is considerable evidence for the association of genetic diversity and fitness (McKay and Latta 2002; Reed and Frankham 2003), the correspondence between molecular markers and adaptive differentiation is unclear (Edmands 2002; Frankham *et al*. 2011). Contrasting genetic divergence with field survival and breeding studies can test the efficacy of marker-delineated provenance zones (Hufford *et al*. 2012), but these studies are time consuming and largely unavailable for most species. An alternative approach is to identify markers with the signature of diversifying selection (Foll and Gaggiotti 2008; Fischer *et al*. 2011; Funk *et al*. 2012). Comparisons can then be made between subsets of neutral and candidate selected markers for the delineation of provenance distance, and also to describe the scale of intraspecific adaptation for environmental variables that drive natural selection (Krauss *et al*. 2013; Stingemore and Krauss 2013; Hamlin and Arnold 2015).

Previously, we examined the consequences of within-population, short- and long-distance crosses (at scales of ∼100 m, 10 km and 100 km, respectively) for early fitness of the plant species *Stylidium hispidum*, endemic to southwestern Australia (Hufford *et al*. 2012). We found evidence for both inbreeding and outbreeding depression among F1 progeny, supporting an intermediate optimal outcrossing distance in this species (Waser 1993; Schierup and Christiansen 1996). At the same time, we compared genetic structure and patterns of gene flow within and among the four populations included in the cross-pollination study. Significant genetic differentiation among populations correlated with an increased risk of outbreeding depression, suggesting that parental divergence corresponded to fitness of intraspecific hybrid progeny (Pekkala *et al*. 2012). Further characterization of spatial genetic structure representative of the species' range is useful to provide greater resolution for the estimate of an optimal distance to minimize population divergence among seed sources in reintroduction programmes.

In this study, we examined molecular marker differentiation among 16 populations of *S. hispidum*, including 4 populations represented in the original study of hybrid fitness (Hufford *et al*. 2012). We analysed genetic diversity and population structure along a north–south transect of the species' range for 134 amplified fragment length polymorphism (AFLP) markers. Our goal was to estimate the distance at which significant differences are likely among potential source and recipient populations in restoration. We also conducted surveys for markers displaying signatures of selection, and examined the relationship of neutral and putatively selected markers with relevant climate variables. As a result, we were able to (i) estimate seed transfer distance within which genetic divergence is low and population fitness less likely to be affected by maladaptation and outbreeding depression and (ii) identify both putative markers and environmental variables linked to fitness differences in populations across the species' range. We discuss our findings in light of seed sourcing for reintroduction of this species as well as implications for the definition of ‘local’ provenance in ecological restoration.

## Methods

### Location and study species

Southwestern Australia is a global biodiversity hotspot with >8000 recognized vascular plant species, of which nearly half are endemic to the region (Myers *et al*. 2000; Hopper and Gioia 2004). Vegetation in the Southwest Australian Floristic Region (SWAFR) is adapted to highly weathered and severely nutrient-impoverished soils within an ancient landscape unaffected by glaciation and large tectonic disturbances (Lambers *et al*. 2014). The climate is Mediterranean and annual rainfall primarily occurs during winter months, with a range of ∼500–1400 mm in native *Eucalyptus marginata* (jarrah) forest. Records indicate that the region has experienced a 17 % decline in precipitation between 1975 and 2011, and the increasing severity of drought reflects higher temperatures as well as rainfall deficiencies (Nicholls 2004; Standish *et al*. 2015).

The family Stylidiaceae includes >240 species that occur mainly in Australia, New Zealand and Southeast Asia (Erickson 1958; Wagstaff and Wege 2002). A majority of those taxa are found in southwestern Australia, a region identified as the centre of triggerplant evolution (James 1979; Coates *et al*. 2003). *Stylidium hispidum* (or white butterfly triggerplant) is endemic to the SWAFR and can be found in the jarrah forest understorey along the Darling Scarp east of the Swan Coastal Plain (Western Australian Herbarium 1998). Plants are herbaceous perennials with a rosette growth form and produce one or more scapes with flowering racemes in spring. Flowers are protandrous and have fused styles and filaments, forming a column that is triggered by insects, resulting in pollen transfer. Early chromosome research determined that *S. hispidum* is diploid (*n* = 14; James 1979), and evidence supports obligate outcrossing (Burbidge and James 1991; Hufford *et al*. 2012).

### Collection sites and sampling

**Figure 1.**
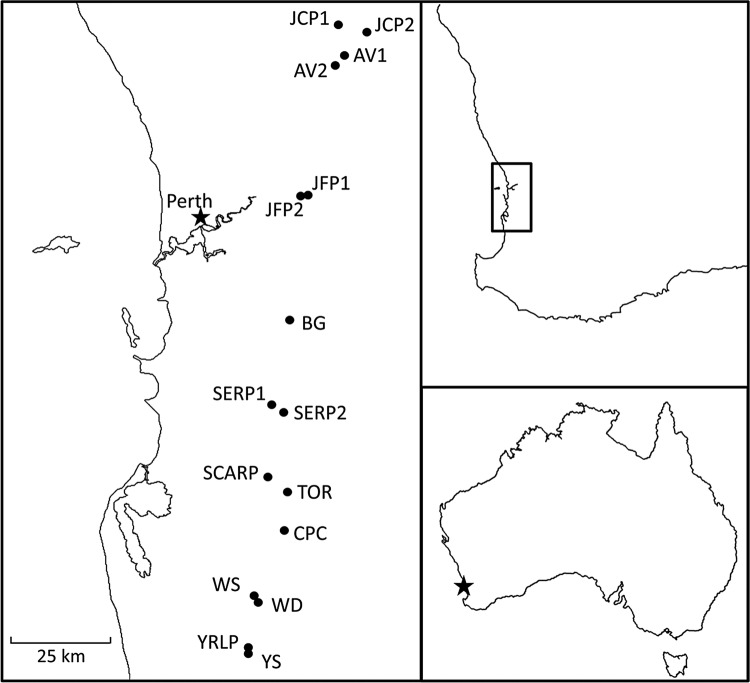
Distribution of 16 sites sampled along a north–south transect of the range of the endemic triggerplant *S. hispidum* in southwestern Australia. Population IDs and descriptions are given in Table 1.

We sampled leaf and bud tissue from 16 sites and a minimum of 30 plants per site along a north–south transect from Julimar Conservation Park to Dwellingup National Forest (Fig. 1). This transect spanned a latitudinal gradient of 160 km, representing much of the species' range. Four of the 16 sites included populations for which we have data describing both inbreeding and outbreeding depression as a result of short- and long-distance crosses in this species (Table 1; Hufford *et al*. 2012). Populations of *S. hispidum* at these sites were found in a patchy distribution on lateritic soils and consisted of 300 or more plants. Tissue for genetic analyses was collected in spring and stored at −80 °C prior to DNA extraction. Plants within each population were sampled an average of 10 m apart to avoid genotypingrelated individuals.

**Table 1.** Sampled locations and genetic diversity indices for 16 *S. hispidum* populations along a north–south gradient, including latitude (N°) and longitude (W°) in decimal degrees, sample size (*n*), the number of locally common markers (ƒ) in ≤50 % of populations, per cent polymorphic loci (PLP) and expected heterozygosity (*H*_e_) with standard errors. ^1^Four populations that exhibit early outbreeding depression in progeny of long-distance crosses (Hufford *et al*. 2012).

Location	Population ID	N°	W°	*n*	ƒ	PLP (%)	*H*_e_	
							Mean	SE
Julimar Conservation Park	JCP1	−31.4907	116.1671	42	6	89.6	0.257	0.016
	JCP2	−31.5076	116.2338	29	5	81.3	0.235	0.017
Avon Valley National Park	AV1^1^	−31.5621	116.1818	33	7	89.6	0.247	0.015
	AV2^1^	−31.5849	116.1595	37	7	83.6	0.251	0.015
John Forrest National Park	JFP1	−31.8904	116.0944	30	7	81.3	0.220	0.015
	JFP2	−31.8911	116.0784	33	8	82.8	0.226	0.016
Bungendore Park	BG	−32.1826	116.0520	32	6	83.6	0.235	0.016
Serpentine National Park	SERP1	−32.3826	116.0096	32	8	91.0	0.238	0.014
	SERP2	−32.4001	116.0388	32	6	88.8	0.243	0.015
Dwellingup State Forest (DSF)	SCARP	−32.5517	116.0022	28	5	79.9	0.200	0.016
	TOR^1^	−32.5860	116.0472	33	6	76.1	0.213	0.016
	CPC^1^	−32.6772	116.0403	32	6	76.9	0.228	0.016
DSF Waroona	WS	−32.8302	115.9706	32	3	79.9	0.221	0.016
	WD	−32.8458	115.9794	32	6	85.1	0.250	0.016
DSF Yarloop	YRLP	−32.9514	115.9562	31	5	76.1	0.221	0.016
	YS	−32.9584	115.9556	30	2	62.7	0.186	0.017
			Mean	32.4		81.8	0.230	0.004

### Genetic analyses

Genomic DNA was extracted according to the methods of Wagner *et al*. (1987) and Byrne *et al*. (2001), and amplification of AFLP markers followed Vos *et al*. (1995) with minor modification (Hufford *et al*. 2012). Two primer combinations, EcoRI-AGG/MseI-CTG and EcoRI-ACC/MseI-CTG, produced distinct bands and were selected for analysis using a Beckman CEQ 8000 Genetic Analyser. DNA fingerprints were scored manually with Beckman fragment analysis software and error rates were calculated at ≤3 % by comparison of one or more duplicate samples for each genotyping run. Fragment analyses were performed for 518 individuals from the 16 sites representing an average of 32.4 individuals per site (Table 1). Amplification of DNA was not successful for all individuals and resulted in a sample size <30 for two populations. Data files consisted of the presence or absence of AFLP bands were prepared for analyses with functions available in GenAlEx 6.5 and AFLPdat software (Ehrich 2006; Peakall and Smouse 2006).

Summary statistics for AFLP data were calculated using GenAlEx and included measures of the proportion of polymorphic loci (PLP) and unbiased estimates of expected heterozygosity (*H*_e_). We conducted regression analysis to test the relationship between population size and estimates of genetic diversity. We also recorded the number of private markers, as well as locally common markers found in <50 % of populations. Differentiation among and within populations (calculated as Φ_PT_, an analogue of *F*_ST_) was estimated using an analysis of molecular variance (AMOVA) implemented in GenAlEx and based on 9999 permutations. We examined the relative genetic dissimilarity among sites using non-metric multidimensional scaling (MDS) ordination in PRIMER 6.1.13 software (Clarke and Gorley 2006) for a matrix of pairwise Φ_PT_ values calculated in GenAlEx.

Bayesian methods allowed investigation of the number of significant genetic clusters represented in the dataset without prior assumptions of population number. Assignment of individuals to population clusters was conducted using methods implemented in STRUCTURE 2.3.3 software with the recessive alleles option for dominant AFLP markers (Pritchard *et al*. 2000; Falush *et al*. 2007). We ran 10 iterations with a burn-in period of 150 000 and 300 000 Markov chain Monte Carlo (MCMC) cycles (University of Oslo Bioportal; Kumar *et al*. 2009) using the default model that infers α and assumes admixture and correlated allele frequencies. The most likely number of clusters represented by the data was determined using the method described in Evanno *et al*. (2005), which calculates Δ*K* as the second-order rate of change of the log probability of the data. In cases where population structure is hierarchical, the method of Evanno *et al*. (2005) only detects significant clusters at the highest level of the hierarchy. Accordingly, we ran STRUCTURE for subsets of the data based on *K* clusters identified in the first run and repeated analyses for each new subset until the number of clusters was either *K* = 1 or very small (Coulon *et al*. 2008). Values of Δ*K* were calculated using STRUCTURE HARVESTER software (Earl and vonHoldt 2012). We subsequently ran CLUMPP (Jakobsson and Rosenberg 2007) to combine results for the 10 runs at each *K*, and results were visualized using DISTRUCT software (Rosenberg 2004).

Pairwise population dissimilarities were investigated via ANOSIM, a non-parametric, multivariate test similar to analysis of variance that calculates R statistics using permutation methods (Clarke 1993; Chapman and Underwood 1999). In ANOSIM, values of each pairwise R statistic are compared with a global test statistic to determine whether populations are significantly differentiated from one another. We tested the significance of pairwise *R* values for a genetic distance matrix representing the 518 individuals and 16 sites using PRIMER and 9999 permutations. Pairwise *R* values were subsequently matched to geographic distance among sites and visualized graphically to determine (i) the minimum distance representing significant genetic divergence for *S. hispidum* and (ii) the global provenance distance inferred from the intercept of the global *R* value with the line of best fit (Krauss *et al*. 2013; Stingemore and Krauss 2013). ANOSIM analyses were then conducted for the pairwise matrix of linearized Φ_PT_ values among the 16 sites to test the significance of clusters derived in Bayesian STRUCTURE analysis.

We tested the assumption that the AFLP dataset represented neutral markers using an approach available in BayeScan V.2.1 software (Foll and Gaggiotti 2008). BayeScan identifies markers with unusually high or low levels of genetic differentiation as outliers that have signatures of diversifying or balancing selection, respectively. Specifically, selection is inferred at an AFLP marker if the marker-specific estimates of *F*_ST_ are needed in addition to population-specific estimates to explain observed patterns of differentiation in the dataset (Fischer *et al*. 2011). The analysis was performed with 20 pilot runs and a 50 000 step burn-in followed by 50 000 iterations and a thinning interval of 10 for the set of polymorphic AFLP markers. Only polymorphic loci were included in the analysis. Outliers were identified at the 1 % significance level, which corresponds to a Bayes factor threshold of ‘decisive’ evidence for selection relative to the neutral model (log_10_ of posterior odds >2; Jeffreys 1961). The false discovery rate (FDR) calculated the expected proportion of false positives for statistically significant results (Foll and Gaggiotti 2008).

### Environmental data and Mantel analyses

To characterize environmental differences, BIOCLIM variables were obtained for each of the 16 sites by extrapolating climate data to the GPS coordinates for each population using DIVA-GIS software (Hijmans *et al*. 2001, 2005). The BIOCLIM dataset includes 19 variables that describe monthly temperature and precipitation patterns for a spatial resolution of ∼1 km^2^ (http://www.worldclim.org/). The sampled area spanned much of the known *S. hispidum* range, which occurs primarily in the high rainfall zone, along a north–south precipitation gradient from ∼700–1200 mm.

To avoid redundancy in environmental data, we first removed variables with high levels of correlation where |*r*| > 0.8 and subsequently conducted principal component analysis (PCA) in JMP 9.0 software. Factor loadings resulting from Varimax rotation were examined to determine the variables with the greatest contribution to the variance in the data (King and Jackson 1999; Graham 2003), and those variables were added to the reduced dataset. Prior to subsequent analyses, data were log_10_(*x* + 1) transformed to improve normality and reduce heteroscedasticity. Dissimilarity matrices of Euclidean distances were calculated among normalized climate variables using PRIMER software. A matrix of geographic distances among sites was generated from GPS coordinates with the SoDA package in R software and also log_10_ transformed (R Development Core Team 2014). Multidimensional scaling ordination was conducted for a similarity matrix of environmental variables among sites.

Correlations among the 16 sites for measures of genetic, environmental and geographic distance were calculated using Mantel and partial Mantel tests in R software with functions in the ‘vegan’ package (Oksanen *et al*. 2013). Mantel statistics were estimated using Pearson's method and 9999 permutations. Spatial structuring of environmental variables can inflate associations unless the effects of geographic distance are removed. Thus, partial Mantel tests were also conducted to determine the strength of the correlation between two distance matrices after removing the effect of a third matrix (Reynolds and Houle 2003). Comparisons of genetic and environmental or geographic distance were made for the full dataset of 134 markers as well as subsets of markers with signatures of diversifying selection, or markers representing neutral genetic variation.

## Results

### Genetic analyses

All but 2 of the 134 AFLP markers were polymorphic and levels of gene diversity were relatively high for the 16 populations with a PLP range from 63 to 91 % (Table 1). Regression analysis detected a significant correlation between sampled population size and per cent polymorphism (*R*^2^ = 0.40, *P* = 0.008) that was not detected for *H*_e_. Locally common markers were identified for each of the 16 populations, but private markers were not present. The AMOVA partitioned 23 % of the total genetic variation among sites and 77 % of the total genetic variation within sites (Φ_PT_ = 0.23; *P* < 0.0001; Table 2).

**Table 2.** Analysis of molecular variance results for 134 AFLP markers representing 518 individuals and 16 sites of *S. hispidum*, and based on 9999 permutations.

Source of variation	df	Sum of squares	Variance components	Variation (%)
Among populations	15	2574.99	4.80	22.66
Within populations	502	8221.85	16.38	77.34
Total	517	10 796.84	21.18	

**Figure 2.**
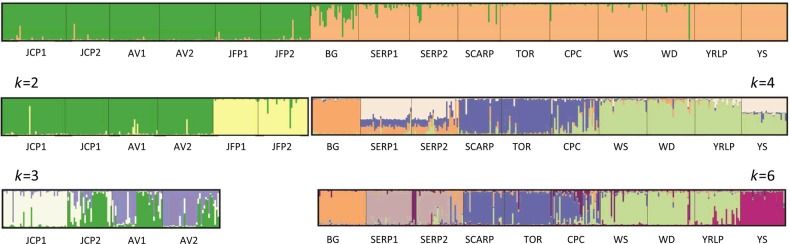
Nested STRUCTURE analyses of AFLP data representing 16 *S. hispidum* collections arrayed from north to south (left to right). Each segment represents one site and each bar is one individual, with shared colour indicating genetic homogeneity among individuals. The first run detected two clusters distributed between the northern and southern range of the species. The second and third tiers of analyses detected a total of either 6 or 10 nested population clusters (where *k* = 10 includes JFP sites).

STRUCTURE analysis of the full dataset assigned individuals to two clusters between northern and southern sites along a border defined by John Forrest National Park (JFP1 and JFP2) and Bungendore Nature Reserve (BG) (Fig. 2). Little admixture was apparent between the two regions. We conducted one or two additional rounds of analysis for each northern or southern cluster. The first STRUCTURE run for the northern sites identified a separate genetic cluster at John Forrest National Park, while the final run separated remaining sites from JCP1 and provided evidence for considerable admixture. Additional runs for the southern region detected four or six population clusters consistent with isolation by distance. Overall, outcomes of nested analyses confirmed the presence of hierarchical population structure with evidence for 6 or 10 distinct population clusters among the 2 regions and 16 sampled sites (Fig. 2).

**Figure 3.**
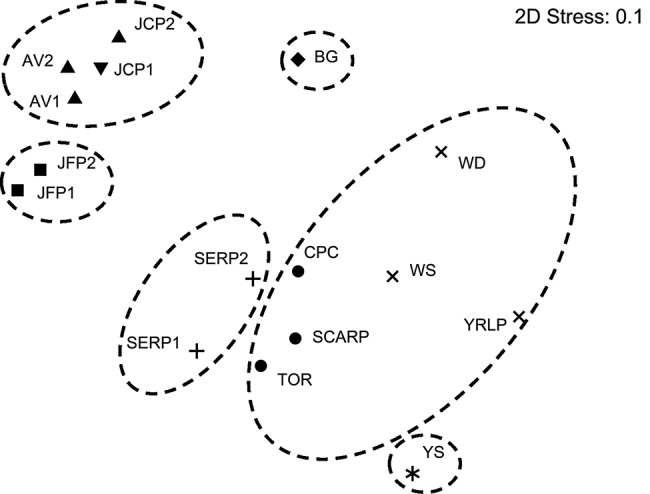
Non-metric MDS results for the matrix of linearized Φ_PT_ values for *S. hispidum* at 16 sites. Stress is an estimate of goodness of fit of the ordination. Ellipses represent significantly different clusters derived in ANOSIM comparisons of STRUCTURE results (where STRUCTURE groups are identified by matching symbols).

Results of ordination analyses reflected the outcomes of Bayesian clustering methods, and indicated a distribution of populations consistent with their geographic distance (Fig. 3). ANOSIM test statistics for the matrix of Φ_PT_ values identified significant pairwise differentiation among the two northern clusters (JFP and JCP/AV sites; *P* < 0.05), the relatively isolated collection at BG and southern sites. However, ANOSIM did not detect significant differences among six of the southern populations. Instead, populations at Serpentine National Park (SERP1 and SERP2) were significantly differentiated from nearby populations in the northern Dwellingup forest (including CPC, Scarp and TOR), and both of these clusters differed from the population (YS) furthest south. ANOSIM, therefore, supported six genetically distinct population clusters similar to the second tier of Bayesian cluster analyses, although population clusters reflected some differences between the two methods.

**Figure 4.**
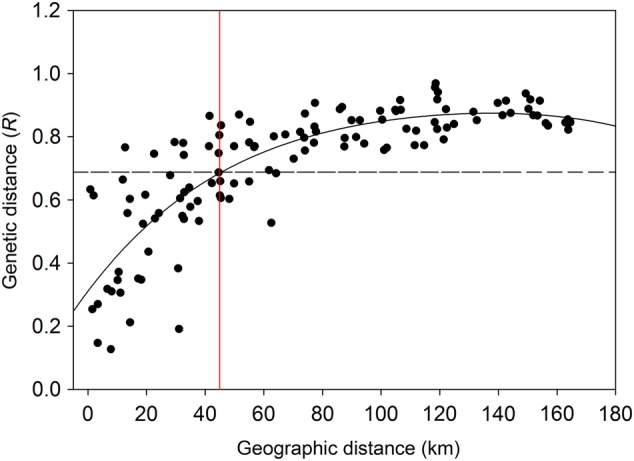
Pairwise *R* values for genetic distance and the best fit polynomial curve among the 518 individuals of *S. hispidum* sampled at 16 locations along a north–south transect of the species' range. The threshold significance of *R* = 0.687 identified a global provenance distance of 45 km (vertical line).

ANOSIM of genetic distance among the 518 individuals and 16 sites generated a global *R* statistic of 0.687 with a significance level of *P* < 0.0001. Of the 120 pairwise combinations, 76 sites were significantly differentiated from one another. Investigation of the geographic separation among sites that had significant pairwise comparisons indicated that the shortest distance between any two sites that were significantly genetically differentiated was 12.7 km (WD and YS in the southern region). All remaining significant pairwise comparisons occurred between sites 22.6 km or more apart, suggesting a minimum patch size of ∼13–23 km (Krauss and Koch 2004). We inferred a global provenance distance of 45 km represented by the point where the global *R* value intersected the line of best fit (Fig. 4). When considering the distance class from 45 to 67 km, ∼44 % of pairwise combinations resulted in an *R* test statistic <0.687, and so were not significantly different. All sites located at distances >67 km apart were significantly differentiated from one another.

**Figure 5.**
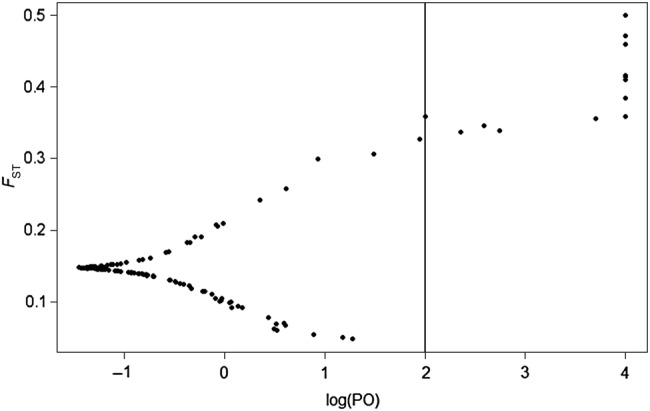
Results of BayeScan outlier analyses for 132 polymorphic AFLP markers amplified in 518 individuals of *S. hispidum* collected among 16 sites. Candidate markers with signatures of genomic selection are represented by points to the right of the vertical line, which indicates the threshold for posterior odds (PO) of 100. Axes plot the marker-specific estimates of *F*_ST_ relative to the log(PO).

BayeScan identified 13 outliers that exceeded the 1 % threshold for selection (posterior odds = 100; Fig. 5) with a FDR of 0.001. All outliers had higher than expected *F*_ST_ values, indicating evidence for diversifying selection, and 9 of the 13 markers were retained in BayeScan analyses when posterior odds were set at 1000. Analysis of molecular variance of the 13 candidate markers reflected greater levels of differentiation (Φ_PT_ = 0.52; *P* < 0.0001) but similar levels of polymorphism (81.7 %) relative to the full dataset (Table 2). The remaining 121 markers fit a model for neutral variation, and AMOVA represented significantly lower levels of differentiation among sites when the 13 selected markers were no longer included in the AFLP dataset (Φ_PT_ = 0.18; *P* < 0.0001).

We ran the ANOSIM analysis a second time using the genetic distance matrix representing the 13 markers with signatures of selection among all individuals for the 16 sites. In this case, 71 of the 120 comparisons were significantly differentiated based on the global *R* statistic (0.737, *P* = 0.0001), and we noted one pair of *S. hispidum* populations that represented significant genetic differentiation at a very short distance (YS and YRLP located ∼0.8 km apart). However, all but two significant comparisons (including WD and YS, 12.7 km apart) once again occurred among sites separated by 23 km or greater, the global provenance distance was ∼45 km and pairs of sites >73 km apart were significantly different in all cases. In effect, the subset of genetic data for outliers replicated results for the full AFLP dataset, but pointed to the potential for sites to differ in adaptive traits at shorter distances than those identified by use of principally neutral marker datasets.

### Environmental data and Mantel analyses

The PCA of climate variables described three factors that explained 96.1 % of the variation in the data. We selected four variables that contributed significantly to factor loadings and had low levels of intercorrelation (Manel *et al*. 2010; Hamlin and Arnold 2015). These variables included the mean monthly temperature range, the mean temperature of the driest quarter (or 13-week period), seasonality of precipitation (defined as the standard deviation of weekly rainfall estimates divided by the mean) and the sum of annual precipitation. Multidimensional scaling analysis resulted in two primary clusters of northern and southern sites, separated by the two populations at John Forrest National Park. Upon examination, the 16 sites represented warmer and drier conditions in the north relative to southern locations, and greater variation in annual temperatures and rainfall at northern or southern margins of the species' range.

Mantel tests indicated a strong correlation in all cases between geographic distance and genetic distance represented by matrices of linearized Φ_PT_ values (*P* < 0.001; Table 3). Simple tests also supported strong correlations between genetic distance and the four climate variables. However, partial Mantel tests for the full 134 marker AFLP dataset and subset of 121 neutral markers only supported a relationship between genetic and geographic distance. This provided evidence for strong spatial autocorrelation of gene diversity among sites. In contrast, the 13 candidate markers were strongly correlated with climate variables, both in simple tests and also when effects of geographic distance were removed in partial Mantel comparisons (*P* = 0.002). At the same time, outliers continued to exhibit a strong correlation with geographic distance when partial Mantel tests removed the effects of climate (Table 3). We subsequently divided climate variables into two subsets reflecting variation in mean temperature or precipitation, and discovered that the relationship between the candidate markers and climate variation was solely the result of correlation with the two variables for annual precipitation (Mantel's *r* = 0.325, *P* = 0.005). The effects of spatial autocorrelation remained strongly significant for outliers in all cases.

**Table 3.** Mantel statistics (*R*_M_) and corresponding *P* values in parentheses for simple and partial tests among matrices of genetic, environmental and geographic distance for AFLP data, including subsets of neutral and putatively selected outlier markers.

Simple Mantel (*R*_M_)		Partial Mantel (*R*_M_)	
134 AFLP markers			
Climate	0.602 (0.001)	Distance removed	0.079 (0.245)
Distance	0.798 (0.001)	Climate removed	0.658 (0.001)
121 Neutral markers			
Climate	0.379 (0.003)	Distance removed	−0.185 (0.951)
Distance	0.669 (0.001)	Climate removed	0.614 (0.001)
13 Outliers			
Climate	0.708 (0.002)	Distance removed	0.375 (0.002)
Distance	0.749 (0.001)	Climate removed	0.494 (0.001)

## Discussion

### Genetic differentiation and seed sourcing

We observed genetic isolation by distance among populations of *S. hispidum* along a north–south transect of the species' range. This pattern is consistent with prior results characterizing genetic structure among 4 of the 16 sampled populations, including evidence of strong differentiation between northern and southern sites (Hufford *et al*. 2012). In our earlier study, significant population genetic divergence at a distance of ∼100 km corresponded to an approximate 6- to 10-fold increased risk of outbreeding depression in intraspecific hybrid progeny at early life stages. Consequently, levels of genetic differentiation reported here provide support for the range-wide application of a provenance zone that corresponds to an optimal outcrossing distance by which outbreeding (as well as inbreeding) depression may be minimized when sourcing seed for ecological restoration of this species (Lynch 1991; Waser 1993).

We noted a sharp disjunction in genetic clustering between northern and southern sites at John Forrest National Park and Bungendore Nature Reserve. The distance between those sites was the second largest span (32.7 km) between any two adjacent populations along the sampled transect, and may indicate a limit for pollen dispersal. The largest span between any two adjacent populations occurred between John Forrest National Park and the northernmost sites (34.8 km; JFP2 and AV2), and this distance was also reflected in results of MDS and Bayesian cluster analysis. Evidence of hierarchical structure suggests that populations are nested within northern and southern regions, and defined by limits of gene flow as well as biotic and abiotic factors that drive adaptation (Evanno *et al*. 2005). Examination of the four climate variables included in analyses detected significant differentiation along a north–south boundary similar to results for genetic markers, and supported regional as well as clinal patterns of environmental variation. This pattern was noted previously for both genetic and climate variation representing the range of *Banksia menziesii* in southwestern Australia (Krauss *et al*. 2013).

Results to describe seed sourcing distance were supported by data from both marker and breeding studies of *S. hispidum* (Hufford *et al*. 2012). Comparisons of the relationship between pairwise Φ_PT_ values and geographic distance determined that significant genetic differentiation may occur at a range as small as 13–23 km for the 16 sampled populations. Similarly, intraspecific hybrid progeny exhibited improved fitness when populations were 3–10 km apart relative to within-population or long-distance (111–124 km) crosses (Hufford *et al*. 2012). In addition, regression of pairwise *R* values and geographic distance identified a global provenance distance between any two populations of ∼45 km. The combined studies suggest a minimum patch size for *S. hispidum* with an average radius no greater than 23 km. These data provide a range-wide, quantitative estimate to assist seed sourcing in restoration, and greatly improve upon general ‘rules of thumb’ (Krauss *et al*. 2013; Stingemore and Krauss 2013).

Molecular marker studies often fail to distinguish neutral and non-neutral variation and, therefore, can only provide indirect evidence for adaptation with limited applied value (McKay *et al*. 2005). The identification of candidate markers and their comparison with neutral marker data, however, is directly relevant for environmental management (Kirk and Freeland 2011; Funk *et al*. 2012). Knowledge of the scale of adaptive variation improves the odds of restoring locally adapted traits and, ultimately, species-level evolutionary potential (Hufford and Mazer 2003). Analysis of putative selected markers in *S. hispidum* confirmed a minimum global provenance distance of ∼45 km for sampled sites. These markers correlated strongly with precipitation variables, suggesting that the calculated seed provenance zone corresponds to the scale of adaptive differentiation for climate drivers. Further testing in the field is warranted, however, to determine whether seed transfer within this distance will maintain population fitness.

### Environmental variation

Approximately 90 % of the AFLP markers characterized in *S. hispidum* were consistent with hypotheses of neutral genetic variation (Reed and Frankham 2001). These markers correlated strongly with geographic distance but were not associated with sampled environmental variables. In contrast, the subset of 13 markers with signatures of selection was highly correlated with environmental as well as geographic distance in partial Mantel tests. This difference supports the hypothesis that climate variation, as well as spatial autocorrelation, drives locally adapted genetic differentiation in this species. We detected significant associations with precipitation but not temperature. Fitzpatrick *et al*. (2008) found that altered precipitation regimes are likely to strongly impact species' distributions in southwestern Australia. Given the significance of rainfall patterns for population genetic differentiation in this species, future restoration of *S. hispidum* may need to draw more heavily from northern populations adapted to drought conditions. In this case, unless evidence supports translocation over longer distances, seed sourcing should maintain local provenance while selecting plant material from drier, northern climates. It is likely that rainfall is not the only driver of adaptive genetic differentiation in this species, and the detection of strong associations between genetic and environmental variation will depend on the variables selected for comparison.

### Defining local provenance

The use of local provenance remains a subject of debate, and composite or admixture collections have been argued to avoid genetically depauperate sources near the restoration site, and also to maximize evolutionary potential in altered environments (Broadhurst *et al*. 2008; Breed *et al*. 2013). In most cases, suitably diverse collections from local provenance zones will meet these objectives, and conserve locally adapted traits as well as maintain genetic variation (e.g. Stevens *et al*. 2015). When necessary, the provenance zone may expand if practitioners note high levels of environmental disturbance, small size of remnant populations and evidence that locally adapted genotypes are no longer best suited for restoration sites (Rice and Emery 2003; Breed *et al*. 2013). Even in these conditions, data would not support transfer of seeds at distances >67 km for this species, the upper limit beyond which all populations were significantly genetically differentiated.

Provenance zones define a collection radius, but do not describe measures to conserve diversity. We noted a strong positive association of sample size and genetic diversity measured as per cent polymorphism among all sites. Therefore, general rules for seed collection representing multiple individuals and populations would still apply (McKay *et al*. 2005; Leimu *et al*. 2006). Genetic variation correlates with fitness in many species (Reed and Frankham 2003), and seed collections should target locally common alleles to maintain regional variation (Marshall and Brown 1975). These collections would depend on prior knowledge of spatial genetic differentiation. In addition, the potential for significant divergence at short distances (e.g. 0.8 km apart in this study) in analyses of selected markers strengthens the argument for habitat matching when combining seeds from multiple locations (Krauss and Koch 2004).

## Conclusions

The debate concerning the definition and efficacy of ‘local’ seed provenance zones will likely continue, particularly in light of changing climate conditions (e.g. Broadhurst *et al*. 2008; Sgrò *et al.* 2011; Breed *et al*. 2013; Havens *et al*. 2015; Prober *et al*. 2015). Our method defining local provenance as the threshold at which geographic distance corresponds to statistically significant genetic distance is promising, and contributes to quantitative rather than qualitative guidelines for ecological restoration (Krauss *et al*. 2013). Moreover, this analysis may meet restoration requirements for a range of relatively pristine to highly degraded sites through identification of the distances at which 50–100 % of populations of target species are genetically differentiated. In highly fragmented landscapes, the risk of reintroduction of sources from long distances can be weighed against the likelihood of population genetic divergence (Byrne *et al*. 2011; Breed *et al*. 2013), and corresponding risks of outbreeding depression. Thus, knowledge of population structure and historical patterns of gene flow will remain a critical component of the restoration practitioner's ‘toolbox’ and, when combined with data for selected markers, may shed light on the factors that define species' distributions and the limits of adaptation.

## Sources of Funding

This study was supported by an Australian Research Council Linkage Grant (LP0669757) and industry partners Alcoa World Alumina of Australia and BHP Billiton Worsley Alumina Pty Ltd.

## Contributions by the Authors

K.M.H., S.L.K., H.L. and E.J.V. conceived and designed the study. K.M.H. and S.L.K. conducted field collections. K.M.H. conducted laboratory and data analyses and wrote the manuscript with S.L.K., H.L. and E.J.V.

## Conflict of Interest Statement

None declared.
